# How Xenophobia Shapes Political Party Support: Evidence from COVID-19 in Canada

**DOI:** 10.1007/s12552-025-09480-y

**Published:** 2025-12-02

**Authors:** Victoria Tan

**Affiliations:** Department of Social Policy and Intervention, University of Oxford, Oxford, UK

**Keywords:** COVID-19, Asian Canadians, Racial politics, Anti-Asian discrimination, Linked fate, Canada

## Abstract

Racialized or ethnically marginalized groups typically have strong loyalties to particular political parties, but can these group loyalties be undermined? In this paper, I investigate whether racist but group-specific political discourse can alter these loyalties by activating a sense of linked fate among those who share a panethnic identity (e.g., as Asian). Using Canadian Election Study data in a quasi-experimental research design, I explore the impact of the highly visible, anti-Asian racism during the COVID-19 pandemic and whether this led to changes in political party support among different Asian ethnic groups, relative to the control, in Canada. I find that Conservative Party support declined more steeply for Chinese respondents than for any other Asian communities after the pandemic, despite Chinese being most likely to vote Conservative pre-pandemic. Therefore, I argue that periods of widespread discrimination can lead people to reject parties that are exclusionary against their group. However, despite their shared vulnerability to discrimination, this rejection of the Conservative party did not occur among all those who are racialized within Asian panethnic identity. Hence, racially hostile but group-specific language can potentially undermine a sense of linked fate and collective political action as a result.

**Supplementary Information:**

The online version contains supplementary material available at 10.1007/s12552-025-09480-y.

## Introduction

Anti-Asian racism soared during the COVID-19 pandemic, as reports of anti-Asian hate crimes rose over 500% in some major Canadian cities, and countless other incidents of verbal and physical harassment went unreported (Levin, 2021; Zhao et al., 2022). The politicization and racialization of the virus, initiated by political elites and then embraced by the public, effectively normalized anti-Chinese sentiment. For example, several papers have attributed the spike in anti-Asian racism in 2020 to US president Donald Trump’s use of the term “Chinese Virus” which explicitly linked Chinese people to the disease (Chan et al., 2022a, 2022b; Cooper et al., 2023; Kim & Kesari, 2021; Nguyen et al., 2021). There are well-known political consequences of this exclusionary rhetoric; racialized or immigrant populations tend to distance themselves from political parties they perceive to be exclusionary toward their group (Lee, 2008). This is not only true in the US, but also in Canada, where contrasting party images in relation to inclusivity continue to influence racialized voters’ party loyalty today (Tolley, 2017).

While COVID-19-related racism online and in political discourse primarily targeted Chinese people, the homogenizing of Asians as indistinguishable led to a ballooning of broader anti-Asian racism. Hence, how have different Asian ethnic groups responded politically to this rise in racism? Social exclusion or discrimination based on one’s racial/ethnic group is theorized to affect one’s vote choice by triggering the salience of that racial/ethnic identity, which then enhances the relevance of that identity in decision-making (Kuo et al., 2017). On a collective level, linked fate theory suggests that individuals who feel as though their life chances are directly tied to those of their group will converge in political action as a group against this exclusion as a form of protection from marginalization (Dawson, 1994; Rogers & Kim, 2023). The predicted outcome of this in the context of COVID-19 racism—how exclusionary rhetoric affects political decision-making—may be less predictable however for a panethnic group like Asian Canadians who may at once possess individual ethnic identities (e.g., Korean, Bangladeshi) and a collective panethnic identity as Asian/Asian Canadian.

As such, the current study leverages the spike in anti-Asian racism over the COVID-19 pandemic period as a natural experiment to causally test the following questions. First, I ask: How did the rise in anti-Asian sentiment during COVID-19 affect political party support among Chinese people in Canada? I hypothesize that Conservative Party support declined the most among Chinese respondents, from the federal elections before 2021 to the 2021 federal election. Secondly, I ask: Did other Asian ethnic voters (i.e., non-Chinese East/Southeast Asians (ESEA) and South Asians) respond similarly to this spike as Chinese voters? If so, I expect a greater convergence in political outcomes among Chinese and non-Chinese ESEA respondents than Chinese and South Asian respondents, given that the former pair were likelier to be conflated and thus victimized in COVID-19-related anti-Asian hate. As such, non-Chinese ESEAs are expected to internalize and react politically to the rise in anti-Asian sentiment more than South Asians during this period.

Using observational data from the Canadian Election Study (2015, 2019, and 2021 waves), and a series of difference-in-differences models, I measure changes in vote choice and political party ratings between Chinese, non-Chinese ESEA, South Asian respondents and Other respondents (which constitute the rest of the sample, largely composed of whites) from the pre-2021 to 2021 federal elections. Based on my findings, I argue that anti-Asian discrimination during the pandemic pushed Chinese Canadians away from the Conservative Party. But this effect was not universal for other Asian ethnic groups, suggesting that panethnic linked fate may not have been a prominent factor Asian Canadian’s political decision-making during the 2021 election.

## Theoretical Overview

### Social Exclusion and Political Party Preference

Extensive evidence demonstrates that racialized minorities do not favour political parties they perceive to be exclusionary towards the group they identify with. In an influential study of this trend for Asian Americans, Kuo et al. (2017) examine why Asian Americans tend to support the Democratic party, despite possessing markers of Republican support. Based on the original work on Social Identity Theory by Tajfel and Turner (1986) and findings from Huddy (2013), Kuo et al. (2017) posit that individuals possess various identities (e.g., based on race, gender) and increasing the salience of one identity can activate preferences or behaviours related to that identity. Through a series of experiments, the authors conclude that the Republican Party’s socially exclusionary rhetoric towards racialized minorities and immigrants pushes Asian Americans away and towards the Democratic Party (Kuo et al., 2017). Consistent with this earlier work, two recent studies find that COVID-19-related anti-Asian racism was associated with increased Democratic Party affiliation and likelihood of voting Democrat in the 2020 presidential election for Asian Americans (Chan et al., 2022a, 2022b).

While the question of how social exclusion motivates political party loyalty among racialized and immigrant communities has been less extensively explored in Canada, a similar trend exists. Non-white immigrant voters have historically tended to support the nation’s centre-left Liberal Party, despite being more socially and economically conservative than their white counterparts who have tended to support the centre-right Conservatives (Bilodeau & Kanji, 2011; Blais, 2005). Scholars reason that the Liberal Party’s role in establishing the Multicultural Act of 1988, which cemented Canada’s image as a multicultural haven, swayed racialized and new immigrant voters to the party (Coloma & Pon, 2017). In the early 2000s however, the Conservative Party found some success in its appeals to Chinese voters, when former Conservative prime minister Stephen Harper issued a formal apology for the party’s role in having established the Chinese Exclusion Act, the first law to ever bar immigration solely based on race/ethnicity (Kwak, 2019). As Kwak (2019) writes, this apology was a key strategy in the Conservative Party’s campaign to sway Chinese voters and ultimately win the 2006 federal election that ended more than a decade of Liberal reign. This historical evidence supports the idea that notions of inclusion matter for Asian voters in Canada; a party that promotes exclusionary rhetoric may be punished politically, while a party that promotes inclusion may be rewarded.

More recent studies, using survey experiments, have studied the importance of ethnic identity or perceived exclusion towards one’s group on political decision-making, primarily with co-ethnic candidate support as the outcome. For instance, Chinese Canadian participants who perceive insults towards their group as personal insults are more likely to support co-ethnic candidates (Goodyear-Grant & Tolley, 2019). The authors conclude that it is the “politicization of [ethnic] identity that motivates affinity”, as voting for “one’s own” is a reaction of positive self-affirmation in response to negative views of one’s group (Goodyear-Grant & Tolley, 2019). Furthermore, Besco (2015) found that Chinese and South Asian Canadians who reported strong identification with their ethnic group were more likely to favour a co-ethnic candidate over a white candidate, compared to those with weaker ethnic affinity. These findings suggest that the politicization of ethnic identity can affect racialized Canadians’ party loyalty, especially among the Asian diaspora in Canada.

### Panethnic Linked Fate

While social exclusion against one’s racial/ethnic group may be politically mobilizing, a question remains with regards to the vastly diverse Asian diaspora: which “group” matters? People of Asian descent are the largest non-white racialized group in Canada, with a plethora of different ethnic or ethno-religious identities, histories, and cultures (19.3% of the whole population; Government of Canada, 2024). Yet, they can also be referred to, or identify themselves collectively under, the panethnic umbrella terms “Asian” or “Asian Canadian”. In her scholarship on Asian American identity, Yen Le Espiritu (1992) first popularized and defined the concept of panethnicity as a “politico-cultural collectivity made up of peoples of several, hitherto distinct, tribal or national origins.” As she writes elsewhere, the development of panethnicity is partly a response to being racialized as “homogenous by outsiders” and thus “panethnic groups are not biologically differentiated groupings but are social, cultural, and legal constructions,” (Espiritu, 2016). In addition, panethnic co-members possess a greater affinity to their shared category when it is used largely in a bottom-up approach (e.g., in solidarity against a shared sense of injustice) as opposed to a top-down one (e.g., when governments create such labels to distinguish populations; Espiritu, 2016; Okamoto & Mora, 2014). As such, panethnicity is a multi-layered process of ethnic identification and ethnic boundary shifting, which enables members to both mobilize under a collective identity and preserve subgroup distinctions (Okamoto & Mora, 2014).

The effects of panethnic identity on political outcomes like partisanship is a burgeoning area of research that relies often on the notion of linked fate (Okamoto & Mora, 2014). Originally developed by Dawson (1994) to understand Black political identity and behaviour, linked fate is defined as the belief that one’s life chances or interests are linked to those of one’s racial or ethnic group. A critical implication is that awareness of a shared oppression leads to a convergence in political action among group members as a rational and efficient form of protection against marginalization (Rogers & Kim, 2023). In this way, linked fate also helps to causally link how broader conditions shape individual-level decisions. For instance, extant racial disparities and ramifications of slavery and Jim Crow-era segregation motivate strong Democratic Party support among Black Americans, regardless of their own socioeconomic position or encounters of systemic racism (Rogers & Kim, 2023). The resulting social expectations of Democratic party support work to reinforce this pattern today (White & Laird, 2020).

Linked fate, as applied to the Asian panethnicity, has proven to be less straightforward than its original utilization to describe the Black American experience. For one, panethnicity involves an inherent tension between possessing a subgroup or distinct ethnic identity while also considering oneself under a collective label (Okamoto & Mora, 2014). As well, for Black Americans descended from enslaved people, original ethnic, tribal, or cultural distinctions among their ancestors had largely been stripped by white enslavers, leading to less prominent intragroup differences today; this legacy of structural racism promotes a sense of unity that fosters linked fate among Black Americans (Rogers & Kim, 2023). In contrast, intragroup ethnic and cultural differences are largely preserved among the Asian diaspora in America and Canada, and often deliberately so by group members.

For these reasons, it might be unsurprising that the literature on Asian panethnic linked fate and political outcomes has produced complicated results. On one hand, data from the US’ largest survey of Asian and Asian Americans reveals that 65% of respondents feel a sense of linked fate with other Asians (Nicholson Jr & Mei, 2023). As well, experiences of discrimination or beliefs that one’s group experiences discrimination enhance feelings of panethnic linked fate (Lu & Jones, 2019; Nicholson Jr & Mei, 2023). Furthermore, Le et al. (2020) found that linked fate among Asian Americans increased after the 2016 election, in part due to Trump’s anti-Asian and anti-immigration rhetoric. On the other hand, discrimination may enhance the salience of individuals’ more specific ethnic identity, rather than their panethnic identity; one experimental study found that national origin identity threats enhanced attachments to that identity, but not Asian panethnic identity (Wu, 2022). Evidence during COVID-19 also suggests that while, on average, Asian Americans exhibited a greater relative increase in Democratic Party support compared to other groups, Chinese Americans exhibited the largest increase, while Vietnamese Americans—who have historically leaned more Republican—did not exhibit such a change (Chan et al., 2022a, 2022b). Hence, group discrimination can, but might not always, engender a similar political response among panethnic co-members.

Comparably less is known about panethnic linked fate among Asian Canadians, though historical and qualitative evidence point to the social and political importance of this panethnic identity. Initially, during the 1800s and early 1900s, Asian migrants to Canada maintained distinct identities to avoid group-specific stigmas that arose from political elites and members of the public who preferred a whiter Canada (Chakraborty, 2022; Coloma, 2013; Li, 2007; Ward, 2002). However, as immigration from other Asian countries grew, and disparate Asian ethnic groups became homogenized, racialized, and criticized as “Orientals”, so did a shared sense of oppression—and thus a recognized need for collective organization. The expansion of voting rights to all Asians in Canada in 1948 which enhanced their political participation, and the growing Asian American movement in the US, eventually paved the way for Asian Canadians organize around this identity in politics and culture in the 1970s (Li, 2007; Poojary, 2024).

COVID-19 anti-Asian racism is but one contemporary manifestation of this shared oppression and thus suggests the potential for a collective or aligned political response during this time. As this racism initially targeted Chinese people, many victims of racist verbal and physical attacks were Chinese. However, members of other Asian ethnic groups were similarly targeted. In a survey of more than 35,000 Canadians in 2020, Statistics Canada found that 64% of Korean participants reported having experienced discrimination, along with 60% of Chinese, 52% Southeast Asian, 47% Filipino, 39% South Asian, and 34% Japanese (Government of Canada, 2020). The high rates of discrimination against non-Chinese East/Southeast Asian groups suggests that many of these instances likely took the form of “mistaken identity” hate crimes. This is a frequent form of anti-Asian violence, Espiritu, (2016) writes, as perpetrators are unable to distinguish Asian people phenotypically. Hence, anti-Asian violence, even if initially focused on one group (e.g., Chinese), can affect members of other Asian communities and thus foster “counter-organization at the pan-Asian level” (Espiritu, 2016).

Canada’s Asian diaspora is diverse however, and not all who are considered “Asian” by the state necessarily see themselves, or are seen, as belonging to this identity, which complicates the pathway from linked fate to political action. For example, “Asian” in North America is often used, colloquially and in academic work, to refer to East Asians, and sometimes Southeast Asians. South Asians in contrast are less often included in this group, and thus more often to be excluded from discussions of anti-Asian racism (Day, 2008; Lee & Ramakrishnan, 2020; Yu, 2016). This was indeed the case during COVID-19 as anti-Asian racism was largely seen as affecting East Asians, not South Asians. For example, in a study of Reddit posts among the North American South Asian diaspora, one quoted comment says “…[it’s] huge against East Asians right now, but definitely not us. Although we are all Asians, we do not look the same as them,” (Poojary, 2024). While this commenter suggests that South Asians are Asian, the lack of a perceived threat of violence indicates a lack of linked fate, and perhaps a perception of “Asian” as a nominal category more than a political identity. However, other comments called for greater solidarity among the Asian community, pointing out similarities as targets of shared stereotypes (e.g., the model minority myth) which itself is indicative of linked fate (Poojary, 2024).

As such, the political consequences of panethnic linked fate, especially in response to group-specific racism that harms and conflates other ethnic groups, requires investigation among Asian Canadians. Historical evidence of political organizing around Asian Canadian identity and common experiences of discrimination during COVID-19 point to the potential for a unified political response, especially among Chinese and East/Southeast Asians. The picture is more complicated for South Asians however, whose lower perception of threat of COVID-19-related racism and relative exclusion from Asian panethnicity might impair group solidarity.

### Hypotheses

Hence, in this study on how anti-Asian racism during the COVID-19 pandemic impacted the political party support of Chinese, non-Chinese East/Southeast Asians (ESEAs), and South Asians (SAs) in Canada, I pose two hypotheses:

#### Hypothesis 1

Conservative Party support will decline from the pre-2021 to 2021 federal elections among Chinese people relative to Others (the remaining sample), while support for the Liberal Party and NDP will increase.

#### Hypothesis 2

Changes in political party support will be more similar among Chinese and non-Chinese ESEA voters than among Chinese and South Asian voters.

## The Case of Anti-Asian Racism During COVID-19 as a Natural Experiment

This paper utilizes the surge in anti-Asian racism over the COVID-19 pandemic period as a natural experiment to test the effects of racist but group-specific political discourse on political party preferences among those who share a panethnic identity with the targeted group. The theoretical mechanism behind the paper’s hypotheses—that Asian, primarily Chinese, Canadians would distance themselves from the Conservative Party because of the Party’s association with COVID-19-related anti-Asian racism—rests on two main assumptions: First, that anti-Asian racism during this period was pervasive and sudden enough to act like a “shock” to trigger the salience of panethnic or ethnic identity among Asian Canadians; and second, that Asian Canadians were wary of the Conservative Party’s insensitivity to, or complicity in, this racism. These assumptions are expanded on below.

### COVID-19 Racism as a Shock to Trigger Ethnic Identity Salience

The COVID-19 pandemic has often been referred to as a time of “dual pandemics” to highlight the parallels between the sudden spread of the virus and the surge in anti-Asian racism (Yeh et al., 2022). Once Wuhan, China was identified as the virus’ geographic origin, right-wing politicians and their supporters hastily linked the disease to Chinese people in harmful ways, inciting a blatant revival of “Yellow Peril” discourse—the longstanding belief and fear that Asians are threats to the Western world (Kim & Kesari, 2021; Li & Nicholson Jr., 2021). Like SARS-Cov-2, anti-Asian sentiment during this period was borderless, vectored by a highly globalized media system that legitimized these imagined anxieties (Chen & Wu, 2021). Indeed, while Trump notoriously catalyzed this surge with his use of the term “China Virus”, Asian Canadians have reported in qualitative interviews that this racism was not merely “an American problem”, and that the coverage of US politics has “influenced the crazy people in our country as well” (quoted in Leigh et al., 2022). Unlike the virus however, anti-Asian sentiment was not so effectively inoculated against with an equivalent vaccine.

Evident throughout stories of Asian Canadians’ experiences with discrimination, their worsened mental health, and adjustments to daily routines to avoid potential racist encounters, is the awareness that their existence as Asian was perceived as a larger threat to society than their actual infection or vaccination status (Chakraborty, 2022; Cooper et al., 2023; Korzinski, 2020; Leigh et al., 2022; Lou et al., 2022; Wu et al., 2020). These anxieties reflect a renewed, if not newfound, sense that their ethnic identity is a critical marker for how they might be treated in white-dominated society and thus, their marginalized position; this recognition too, created an urgency to work to unravel these injustices. As one participant shared in Cooper et al. (2023), “if we experience stigma, we should focus on what we should do to fight against it.” Given the thousands that took to the streets to protest anti-Asian sentiment in Canada, this exclusion was evidently effective in priming the salience of ethnic identity and stimulating a political response among Chinese and Asian Canadians (Zhao et al., 2022).

### Canada’s Political Parties and Anti-Asian Racism During COVID-19

Canada’s two largest political parties responded to COVID-19-related anti-Asian racism in distinct ways, ultimately reproducing their historically contrasting images regarding the social inclusion of racialized minorities and immigrants. Liberal politicians denounced the rise in anti-Asian sentiment and leaned into Canada’s multicultural identity, while Conservatives largely remained silent or, in some instances, contributed to the hostility. A particularly prominent case that exemplified the latter involved Conservative Ontario MP Derek Sloan, who publicly questioned whether Canada’s chief public health officer, Dr. Theresa Tam, “work[ed] for Canada or China?” (Chakraborty, 2022; Harris, 2020). In accusing Dr. Tam of letting “Chinese Communist propaganda” to influence her decisions in managing the pandemic, Sloan leaned into “perpetual foreigner” or “foreign spy” tropes, which fanned the flames of Yellow Peril. His insinuation that her loyalty to Canada was questionable because of her Chinese background unsurprisingly received backlash, though Sloan emphasized that his call for Dr. Tam to step down had nothing to do with her ethnicity (Harris, 2020).

While party leaders Justin Trudeau (Liberal) and Jagmeet Singh (NDP) criticized Sloan’s attack on Dr. Tam and denounced the rise in anti-Asian racism in Canada more broadly, Conservative leader Andrew Scheer refused to comment when pressed by reporters (Harris, 2020). Sloan’s hostility and Scheer’s indifference were not the only signs of a Conservative Party less-than-sensitive to the issues of Asian Canadians during the pandemic, however. In the 2021 federal election, the Conservative Party under Erin O’Toole lost to the Liberals, with a notable drop in support in ridings with a large concentration of immigrants and Chinese Canadian voters (White, 2022). One reason, Ellis (2022) suggests, was O’Toole’s “particular obsession with China.” He argues that O’Toole’s positioning of China as “symbolic” of the issues blue-colour Canadians faced, and the Conservatives as the only party willing to “take a firm stance against that country’s transgressions” replicated a strategy that was successful for US and UK conservatives but that this “anti-China rhetoric likely contributed to them losing three seats in metro Vancouver and Toronto” (Ellis, 2022). To use Gao’s (2022) “triple conflation” framing of Sinophobia during the pandemic: O’Toole’s political antipathy towards China, coupled with Yellow Peril notions that Chinese people spread disease and are inherently loyal to China (and hence suspicious, as in the case of Dr. Tam) likely escalated distrust against the Conservatives during this time of peak racial violence against Chinese people.

Canadians are attentive to whether the parties they support appear to have their best interests in mind; for racialized and immigrant voters, protection against marginalization is an important factor in this decision. Regardless of the authenticity with which Canada’s parties commit themselves (or not) to matters of inclusion, it remains evident that Conservatives possess a less favourable image than Liberals in this regard. Though the Conservative Party has made numerous attempts throughout the past few decades to win over primarily Chinese and South Asian immigrant voters, its more traceable xenophobic history mars its image as *the* party for racialized voters (Adams, 2022; Tolley, 2017). As a result, the complicity of Conservative leaders in anti-Asian racism during the pandemic likely pushed away Asian, especially Chinese, voters from the party, in a way that was likely not the case for the rest of Canadians. This study tests for this effect and addresses an additional question of whether other non-Chinese East/Southeast Asian voters were as mobilized against the Conservatives as much as Chinese voters. This study builds on research on social exclusion and political support by evaluating how rhetoric that targets a specific group might influence the vote choice of panethnic co-members. As such, the surge in anti-Asian sentiment over the COVID-19 pandemic period provides a critical and optimal moment to evaluate political consequences of this animus for Asian communities in Canada.

## Methods

### Data and Variables

This study uses individual-level repeated cross-sectional data from the Canada Election Study (CES), the country’s largest and foremost study on electoral and political behaviour. The CES is administered every federal election year and is completed by both Canadian citizens and permanent residents (aged 18 or older) across all of Canada’s provinces and territories. Each survey is a two-wave panel with a campaign-period wave (beginning around a month before election day) and post-election wave (beginning on election day and completed around two weeks after). Only data from the election years 2015, 2019, and 2021 are included in the study, due to limited sample sizes of Asian respondents in the previous survey years. I also limit the sample to those who responded with any of the three main political parties to questions of vote choice, given that this was the case for the vast majority of Asian respondents. Data from the Canadian Election Studies are publicly available (See: Fournier et al., 2015; Stephenson et al., 2024a, 2024b, 2024c).

The final race/ethnicity variable included: Chinese, non-Chinese East/Southeast Asian (ESEA), South Asian, and Other (i.e., anyone who was not one of the three ethnicities; majority of whom werewhite) (Table 1). Asian ethnicity was proxied and coded based on a composite measure of respondents’ self-reported ethnicity or “visible minority” status, country of birth, and language spoken at home or first language. A composite measure was required due to inconsistencies in the way race/ethnicity was probed for across survey years, and because more detailed Asian ethnicity data were required for the study. Open-ended text data were searched for relevant terms and coded accordingly, when one’s ethnicity or language was not listed as an option in the multiple-choice questions (e.g., an individual who responded “Other” to self-reported ethnicity and wrote “Indonesian” in the text box). The proxying process is imperfect given survey question constraints, but examples (non-exhaustive) are as followed: Respondents were coded as Chinese if they spoke a Chinese language (e.g., Mandarin, Hokkien, Cantonese), reported their ethnicity as such, and/or came from a Chinese-speaking country. non-Chinese East/Southeast Asian respondents were those from countries like Vietnam and Japan who also were not previously coded as Chinese (i.e., a person who self-identifies as Korean but was born in China would be assumed to be Korean and counted as such); and South Asians were those from countries like India, Sri Lanka, or Bangladesh and/or who also reported speaking languages like Urdu or Tamil.

**Table 1:** Size of analytical sample by race/ethnicity and survey year, Canada Election Study 2015–2021

	Pre-2021 period		2021 period	Overall
	2015	2019	2021	
	(*N* = 7203)	(*N* = 24,732)	(*N* = 14,158)	(*N* = 46,093)
Chinese	177 (2.5%)	1144 (4.6%)	541 (3.8%)	1862 (4.0%)
Non-Chinese East/Southeast Asian	92 (1.3%)	789 (3.2%)	592 (4.2%)	1473 (3.2%)
South Asian	175 (2.4%)	1128 (4.6%)	542 (3.8%)	1845 (4.0%)
Other	6759 (93.8%)	21,671 (87.6%)	12,483 (88.2%)	40,913 (88.8%)

To measure federal political party support, I use vote choice in the 2015, 2019, and 2021 federal elections as the primary outcome, and party ratings (0, cold to 100, warm) as the secondary outcome. As many Asian respondents were Permanent Residents and therefore legally unable to vote, the intended vote choice of these respondents was included in the vote choice variable. Party ratings, also known as feeling thermometers, were measured as a continuous outcome on a scale from 0 (really dislike) to 100 (really like). For example, in the 2019 web-based survey, respondents were asked, “How do you feel about the federal political parties below? Set the slider to a number from 0 to 100, where 0 means you really dislike the party and 100 means you really like the party.” A value of 50 is generally considered as feeling neutral toward a party. Party rating is a useful outcome for this study as it involves a more emotional or instinctive response to the parties and might be more susceptible to change than vote choice.

I control for standard sociodemographic variables that are associated with political party affiliation and vote choice, including: age, gender (male, female), immigrant status (yes, no), pre-tax total annual household income (less than $30,000, $30,000–60,000, $60,000–90,000, $90,000–110,000, more than $110,000), labour force status, (employed, unemployed, not working, retired, other), highest education attained (some high school or less, high school degree, undergraduate, postgraduate), and the importance of religion (not at all, not very, somewhat, very). Individuals with missing data or who reported “Don’t Know” to questions about education, income, employment, and immigrant status were removed which eliminated 869 respondents, or 1.9% of the sample and did not lead to changes in the statistical results (Table Supp 4).

### Statistical Analysis

To examine the political consequences of rising anti-Asian racism during the COVID-19 pandemic period, I compare the differences in vote choice and party ratings between Asians and Others from the 2015 and 2019 elections (collapsed into a pre-2021 period) to the 2021 election. Others are the control group in this case. A difference-in-differences modelling strategy is beneficial for this comparison as it enables causal interpretation, given that key assumptions are met. Vote choice is treated as a binary variable, leading to three outcome variables in this category (e.g., voting Liberal or not), and each party rating is also a separate outcome variable (e.g., rating of the Liberal Party from 0 to 100), leading to six total outcome variables. For each outcome, I estimate OLS difference-in-differences (DiD) with the following specification:

$$Y={\beta }_{0}+{\beta }_{1}Race+{\beta }_{2}Period+{\beta }_{3}\left(Race\times Period\right)+{\beta }_{z}Covariates+{e}_{vote},$$

where $Y$ is the outcome of interest, $Period$ indicates whether respondents were surveyed pre-2021 or 2021, and $Race$ indicates belonging to the Treatment (either Chinese, non-Chinese ESEA, or SA), or the Control (Other) ethnicity categories. Among the coefficients, ${\beta }_{0}$ is the constant, or the estimated outcome value for Others Pre-2021; ${\beta }_{1}$ estimates the change in $Y$ between the Treatment and Control groups; ${\beta }_{2}$ estimates the change in $Y$ across time periods for Others; ${\beta }_{Z}$ estimates the contribution of the control variables; and ${\beta }_{3}$ is the DiD estimator of interest which indicates the difference in changes in $Y$ between the Treatment and Control groups from pre-2021 to 2021:

$${\widehat{\beta }}_{3}=\left({\overline{Y} }_{Chinese,2021}-{\overline{Y} }_{Other,2021}\right)-\left({\overline{Y} }_{Chinese,Pre-2021}-{\overline{Y} }_{Other,Pre-2021}\right)$$

For instance, the DiD coefficient indicates the estimated difference in the change in the probability of voting Conservative or not after the COVID-19 period between Chinese and Other respondents. Because there are three Asian ethnicity groups considered as Treatments, there are three Treatment–Control pairs: Chinese vs. Others, non-Chinese ESEA vs. Others, and SA vs. Others. Standard errors are clustered around the period variable to err on the side of more conservative estimates (Brewer et al., 2018).

### Sensitivity Analysis and Assumption Checks

**Fig. 1:**
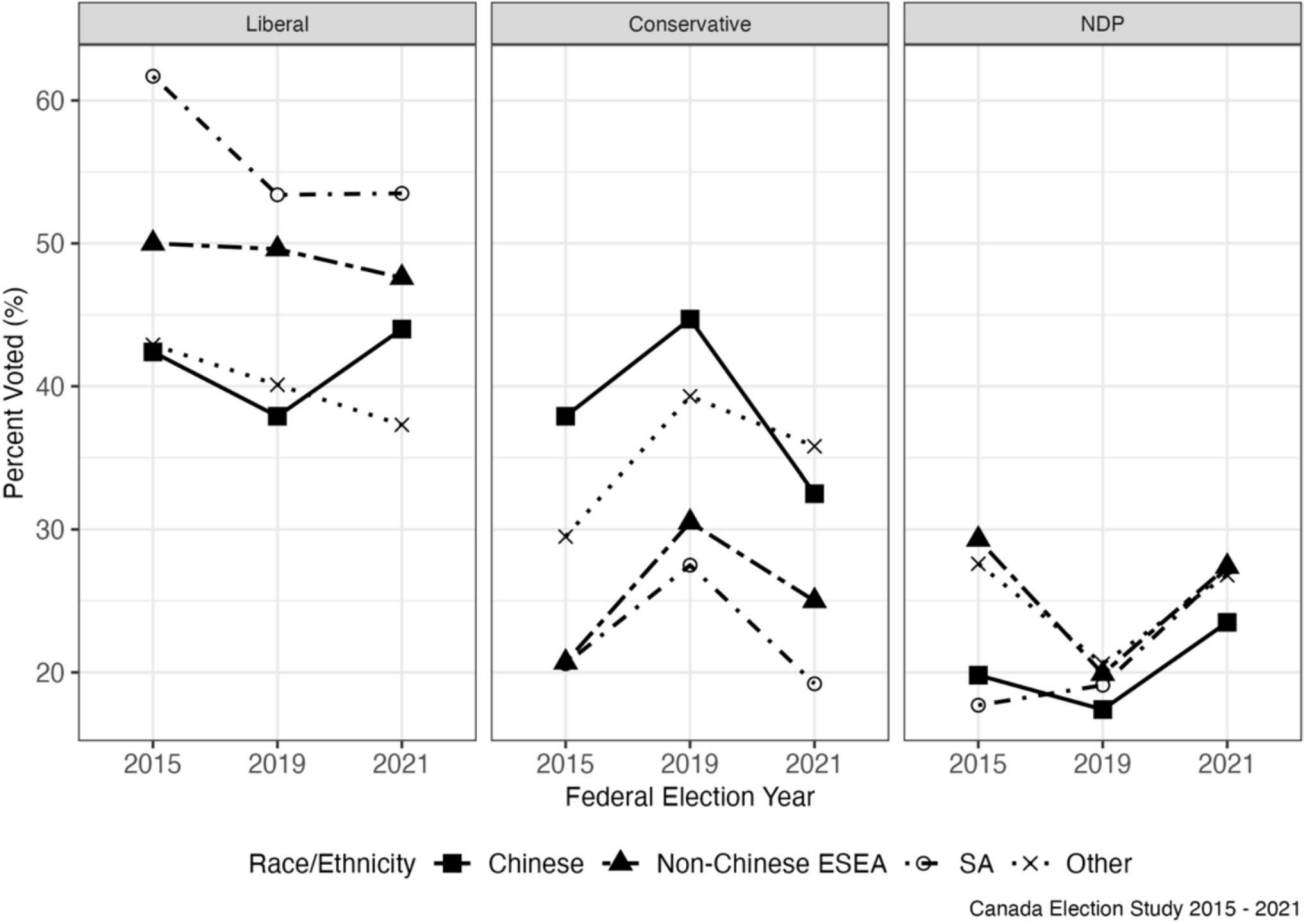
Vote choice by race/ethnicity for each federal election year, CES 2015–2021

**Fig. 2:**
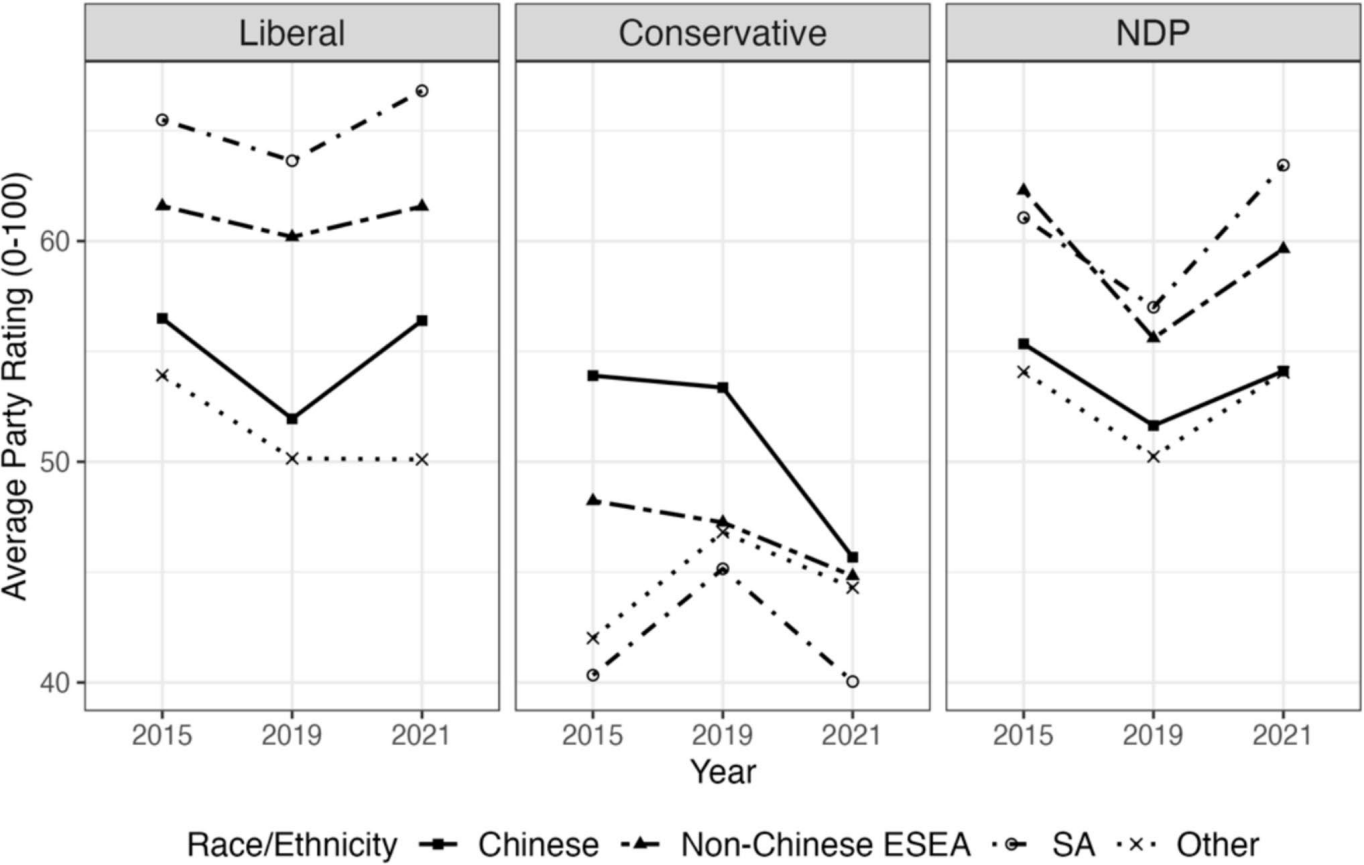
Average ratings for each federal political party, by race/ethnicity and federal election year, CES 2015–2021

To obtain unbiased estimates, DiD relies on several assumptions. The first is the parallel trends assumption, or the idea that the treatment and control group outcomes would have followed a similar trend over time had the intervention not occurred. As such, the identification strategy relies on the assumption that in the absence of the spike in anti-Asian racism over the COVID-19 period, that political party support among the Asian subgroups would change at a similar rate to that of Other respondents, or the rest of Canada. Trends appear consistent in a visual assessment of vote choice and party ratings for each ethnicity subgroup across the 2015, 2019 and 2021 survey years which provides evidence for this assumption (Figs. 1 and 2).

There are also additional theoretical and methodological considerations regarding the use of the COVID-19 pandemic as a shock period. The first is that economic recession during this period may have influenced people’s redistributive policy preferences which might influence vote choice. I estimate whether these preferences changed over the pandemic in another DiD model and conduct a mediation test to assess whether supporting more redistribution accounts for changes in people’s vote choice over the pandemic period. I treat redistributive preferences (asked on a Likert scale) as a binary outcome (“more redistribution” versus all other responses). The question was only asked in the post-election survey waves, and so these analyses were only conducted on half of the total sample.

Additional analyses were done for vote choice utilizing standard errors clustered around treatment group rather than time as there could be treatment-level unobserved confounders not accounted for with time-level clusters. As the estimates with treatment-level clustered standard errors are less conservative, and the overall results remain the same, I opt for the original time-level clustered standard errors (Table Supp 3).

Furthermore, due to inconsistencies in the way ethnicity and self-identified racial group are asked across survey years, I check the effects of collapsing different groups to form one Chinese ethnicity in my proxying process. It is imperative to note that for the purposes of the study, I treat “Chinese” as a single group for feasibility and parsimony in my analyses. However, there is not one single Chinese ethnicity, but rather over fifty, with the most prevalent group being the Han Chinese which comprise over 90% of the populations in mainland China, Taiwan and Hong Kong (Goodkind, 2019). Importantly too, many from Taiwan and Hong Kong seek to separate themselves from the country of China and Chinese identity, due to long histories of colonialism and contemporary political conflict. As such, I examined the impact of collapsing these various groups by conducting separate DiD models for those within the proxied “Chinese” group who were born in China, and the rest who were not. I find very similar results in the magnitude and direction of the DiD estimates and as such, I utilize the original, collapsed Chinese subgroup.

## Results

The DiD coefficients which estimate the effect of the spike in anti-Asian racism on party support for Asian respondents and the rest of the sample, as well as baseline differences pre-pandemic, are displayed in Table 2 (vote choice) and Table 3 (party ratings). To start, compared to Other respondents, the probability of voting Conservative was 10.8%-points higher for Chinese, but 4.3%- and 6.1%-points lower for non-Chinese ESEAs and South Asians, respectively, in the pre-2021 federal elections. However, after the pandemic, Chinese respondents exhibited the most rapid decline in Conservative Party support; the probability of voting Conservative after the pandemic was lower by 8.27%-points among the Chinese compared to Others. Consistent with these findings, Chinese respondents also exhibited the largest decline in the change in Conservative Party ratings when compared to Others (− 5.2 points on a 100-point scale; 95% CI − 5.48, − 4.93; see Table 3). In contrast, Chinese respondents’ predicted probability of voting Liberal after the pandemic increased by 8.87%-points compared to Others. They were also the only group to exhibit an increase in Liberal voting over the pandemic period. Regarding NDP support, the trend did not change as drastically for Chinese compared to Others however, and the DiD estimate of a 0.6%-point difference between Chinese and Others was statistically insignificant (Table 2).

**Table 2:** Predicted probabilities of voting for each of the three main political parties, Canada Election Study 2015–2021

	Conservative	Liberal	NDP
*Difference-in-differences: change in differences after pandemic*			
Chinese vs. other	− 0.0827* (0.0021)	0.0887* (0.0021)	− 0.006 (0.0043)
Non-Chinese ESEA vs. other	0.0041* (0.0009)	0.0168 (0.0083)	− 0.0209 + (0.0092)
South Asian vs. other	− 0.0349* (0.0003)	0.0245* (0.0063)	0.0104 (0.0065)
*Pre-pandemic differences:*			
Chinese vs. other	0.1077* (0.0052)	− 0.0490* (0.0076)	− 0.0587* (0.0128)
Non-Chinese ESEA vs. other	− 0.0432* (0.0067)	0.0726* (0.005)	− 0.0294 (0.0116)
South Asian vs. other	− 0.0610* (0.0062)	0.1136* (0.0058)	− 0.0526* (0.012)
*Constant*	0.379* (0.0078)	0.402* (0.0040)	0.219* (0.0038)

Standard errors in parentheses, *ESEA* East/Southeast Asian

*P < 0.001, + *P* < 0.05

**Table 3:** Predicted party ratings for the three main political parties, Canada Election Study 2015–2021

	Conservative	Liberal	NDP
*Difference-in-differences: change in differences after pandemic*			
Chinese vs. other	− 5.20* (0.14)	3.95* (0.0005)	− 2.56* (0.077)
Non-Chinese ESEA vs. other	1.73* (0.14)	1.01* (0.166)	− 2.82* (0.1)
South Asian vs. other	− 1.23* (0.094)	2.23* (0.2)	0.39* (0.047)
*Pre-pandemic differences:*			
Chinese vs. other	9.35* (0.331)	− 3.18* (0.212)	− 2.73* (0.201)
Non-Chinese ESEA vs. other	2.47* (0.352)	0.545* (0.099)	1.78* (0.247)
South Asian vs. other	0.448 (0.371)	8.68* (0.094)	2.68* (0.211)
*Constant*	46.7* (0.238)	50.7* (0.149)	50.2* (0.317)

Standard errors in parentheses; Party ratings are on a 100-point scale

*ESEA* East/Southeast Asian

*P < 0.001

**Fig. 3:**
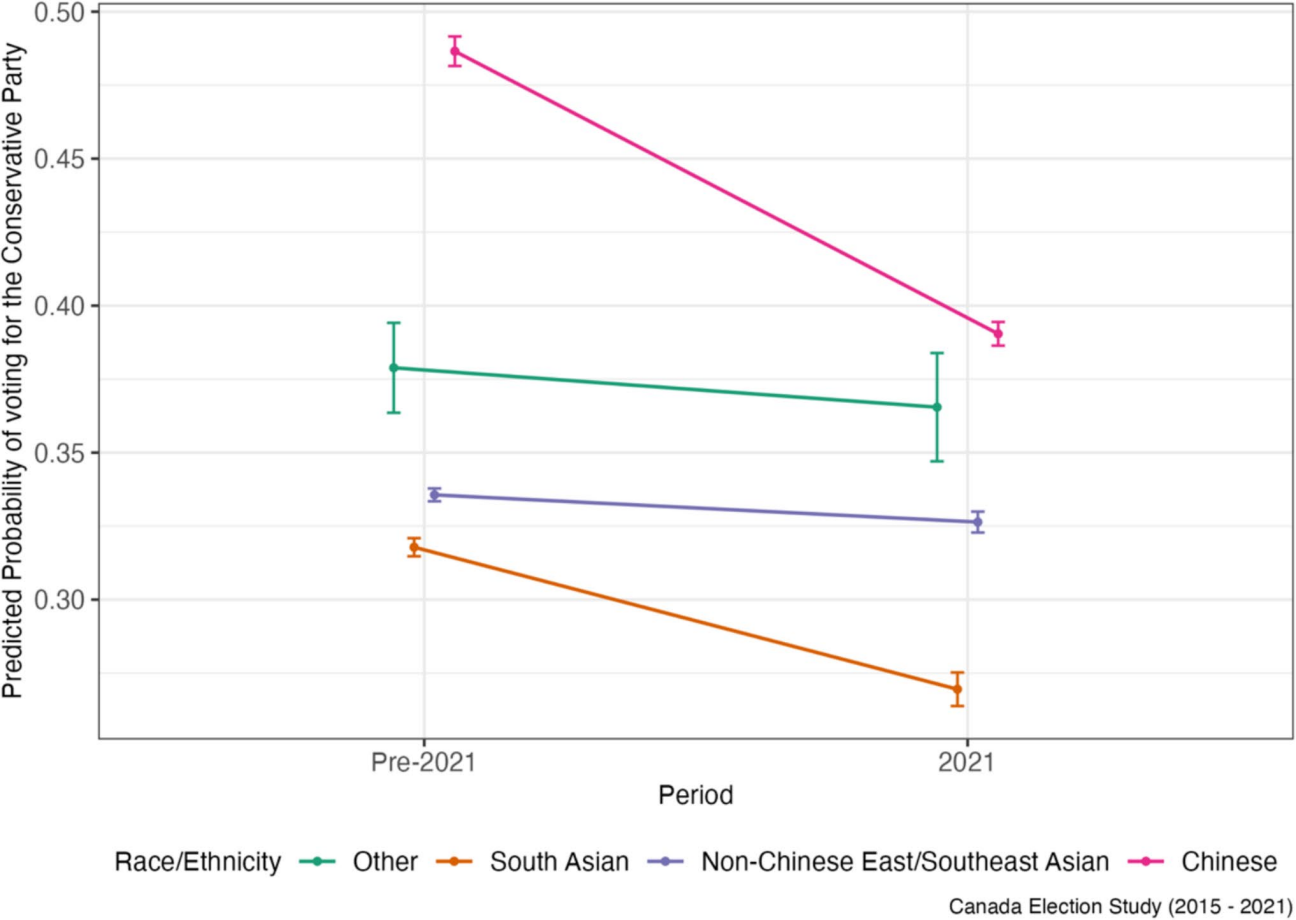
Predicted probability of voting for the Conservative party by race/ethnicity

**Fig. 4:**
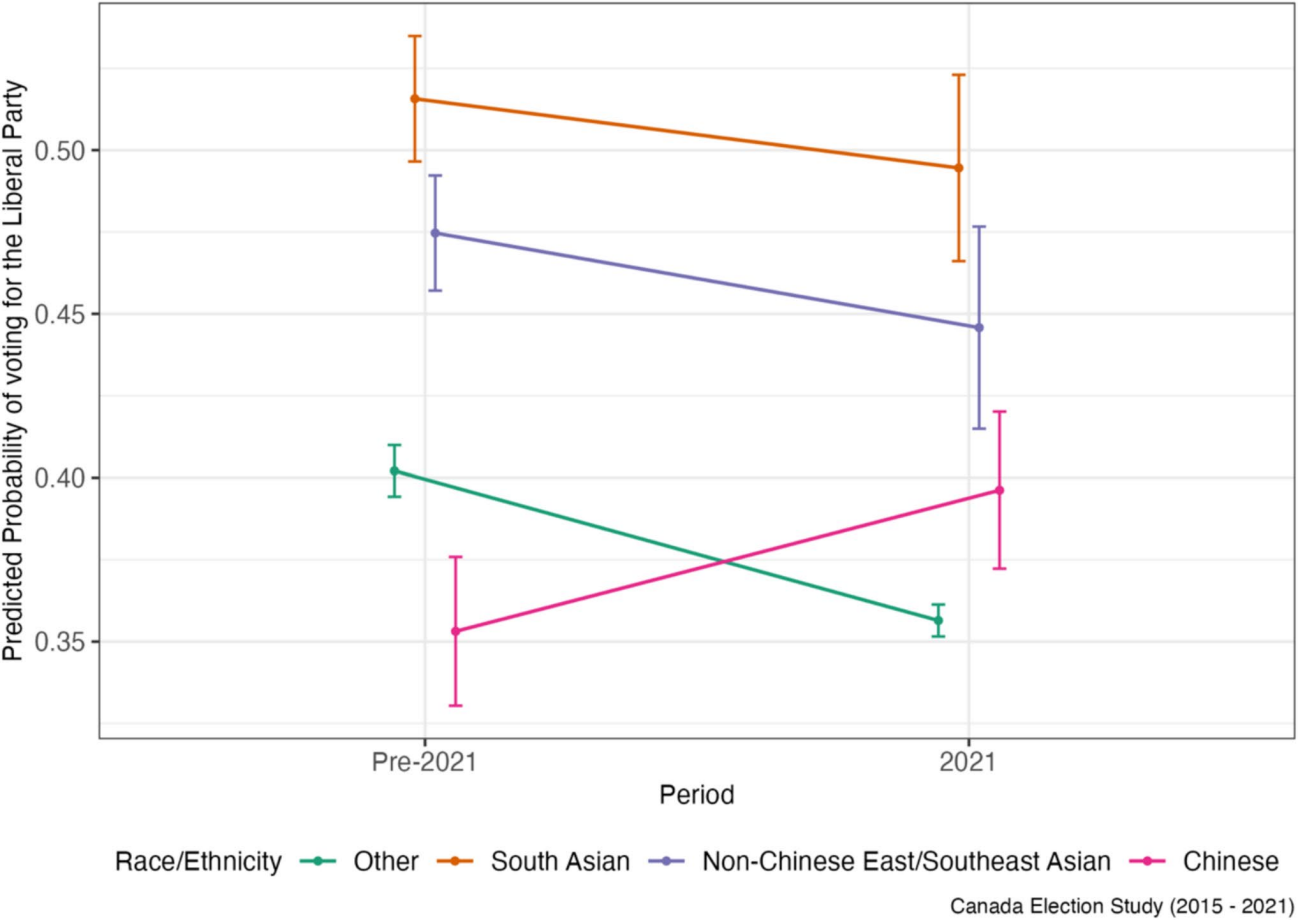
Predicted probability of voting for the Liberal party by race/ethnicity

**Fig. 5:**
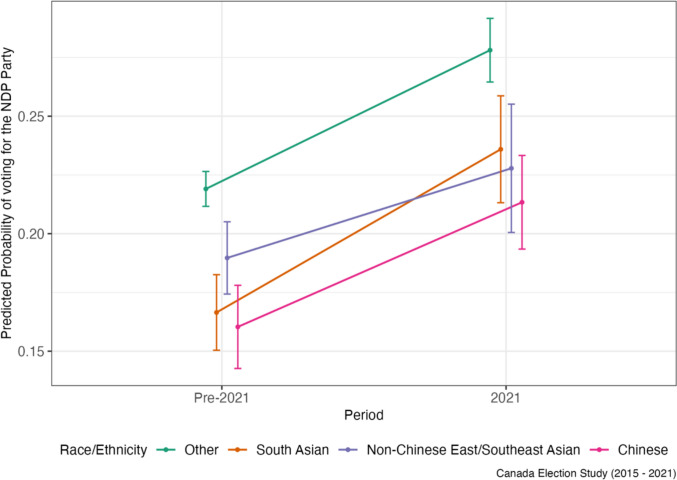
Predicted probability of voting for the NDP by race/ethnicity

For non-Chinese East/Southeast Asians, the predicted probability of voting Conservative declined very slightly over the pandemic, but this decline was less steep than for Others, leading to a slight relative increase by 0.41%-points (Table 2). A similar pattern exists for Conservative party ratings as well, as non-Chinese East/Southeast Asians showed relative increase on average by 1.73 points (Fig. 3), compared to Others, over this time period (Table 3). The relative predicted probability of voting Liberal also increased slightly for non-Chinese East/Southeast Asians, as their decline in support for the Liberals was not as steep as for Others, though the DiD coefficient was statistically insignificant (Fig. 4). A similar pattern is mirrored in Liberal Party ratings, as non-Chinese East/Southeast Asians exhibited a relative increase in their ratings of the party over the pandemic compared to Others (Table 3). At the same time, like for Chinese respondents, NDP support increased over time (Fig. 5) though this increase was not as high as for Others, leading to a relative decline in NDP support for non-Chinese East/Southeast Asians compared to for Others (Tables 2 and 3).

The trends in party support and DiD coefficients were more similar directionally for South Asians to those of Chinese respondents. The predicted probability of voting Conservative declined for South Asians over time by 3.49%-points compared to Others, though South Asians already possessed lower Conservative support pre-pandemic. South Asians also exhibited an increase in the predicted probability of voting Liberal over the pandemic by 2.45%-points relative to Others (Table 2), and an increase in Liberal party ratings by 2.23 points (Table 3). Unlike other Asian subgroups however, South Asians were the only group to possess a relative increase in NDP ratings, and the predicted probability of voting NDP (though this was statistically insignificant).

### Sensitivity Analysis

Given that the spike in anti-Asian racism over this period may not be the sole driver of changes in vote choice, I conduct additional analyses to examine whether economic concerns, a major issue during the pandemic, may have influenced vote choice over this period. First, I conducted the same DiD models as above but with the outcome as the likelihood of reporting “more” support for redistribution (over “less” or “about the same”), a binary variable. At baseline, redistributive preferences were lowest among the Chinese and highest among South Asians (Figure Supp 1). After the pandemic, the probability of reporting more support for redistributive preferences increased the most among the Chinese and to a lesser extent, among Others, but declined for non-Chinese ESEAs and South Asians. According to the DiD results, the difference in the probability of reporting more support for redistribution after the pandemic between the Chinese and Other respondents was 5.49%-points (Table Supp 1). To test how much these changes in redistributive preferences affected vote choice, I then conducted a mediation analysis. The proportion of the total effect of race on vote choice mediated by redistributive preferences was only 8.8% which was not statistically significant (Table Supp 2). I ran another model to test whether redistributive preferences mediate the relationship between income and voting, and I found that the proportion becomes much higher, at 26%-points, which is expected. Thus, I can be more certain that redistributive preferences did not play a large role in affecting racial differences in changes in vote choice over the pandemic. This provides more confidence that the spike in anti-Asian racism had an effect instead.

I also utilize Rambachan and Roth (2023)’s approach for measuring violations to parallel trends with the *HonestDiD* package in R. Their method provides a statistical test of how restrictive the assumption of perfect parallel trends must be. I specify the relative magnitudes restriction in a comparison of Chinese and Others for Conservative vote choice. The *M* or “breakdown” value is relatively small at 0.1, which suggests that violations of parallel trends in 2021 can be up to 10% of the violation of parallel trends pre-2021 (Figure Supp 8). Their method is more ideal for event-study estimation setups however, and so I interpret this as a moderate sensitivity to parallel trends. To further determine whether bias from pre-trend violations affects the original difference-in-differences estimates, additional analyses were done using only 2019 and 2021 data (Figure Supp 9 and Figure Supp 10). The conclusions from the original models still hold when 2015 estimates are removed, which suggests that bias induced from pre-2021 trends are minimal.

## Discussion

Over the COVID-19 pandemic, political elites, and the public soon after, spread racist beliefs about Chinese and Asian people, beliefs once considered dormant. Given the varied responses that Canada’s main political parties had in the face of surging anti-Asian racism, this study sought to examine whether party support among Asian Canadians was impacted as a result. In a difference-in-differences analysis, I evaluate how this heightened period of anti-Asian racism had affected the vote choice and political party ratings among Chinese, non-Chinese East/Southeast Asians, and South Asians, across the pre-2021 to 2021 Canadian federal elections, using Other respondents (remaining sample) as a control.

After the pandemic, Conservative Party support, whether measured through the predicted probability of voting Conservative or through Conservative Party ratings, declined more sharply for Chinese respondents than any other group. This evidence supports the study’s first hypothesis that Chinese Canadians would distance themselves from the Party based on its image as less inclusive and its perceived complicity in the spread of anti-Asian racism during the pandemic. This decline in Conservative support is also noteworthy given that Chinese respondents had the highest predicted probability of voting Conservative prior to the pandemic, even when compared to Other respondents who were mainly white; this heightened pre-pandemic Conservative support may have been due to the Party’s concerted efforts in winning over Chinese voters specifically in first decade of 2000 (Kwak, 2019). In contrast, Conservative support remained largely stable from pre-2021 to 2021 for Other respondents. As such, how Canada’s parties address anti-Chinse racism is likely an important and distinguishing factor in how Chinese Canadians vote.

This study also parses out changes in party support over the pandemic by other non-Chinese Asian ethnic groups, namely East/Southeast Asians and South Asians, to evaluate whether this rise in racism was just as politically mobilizing for panethnic co-members. Contrary to the prediction in Hypothesis 2, this study found that Conservative support remained largely stable for non-Chinese East/Southeast Asians, which translated to a relative increase in support compared to Others from pre-2021 to 2021. In contrast, South Asians exhibited a decline in support, more similar to Chinese respondents. This suggests the rise in anti-Asian racism, which conflated Chinese and East/Southeast Asian groups, did not push the latter away from the Conservatives as it did the former. One interpretation is that exposure to anti-Asian racism may not necessarily manifest in a similar political response among panethnic co-members if a particular group is singled out (i.e., Chinese people). For example, a working paper by Kim et al. (2024) finds that Asian Americans’ linked fate increases after exposure to anti-Asian racism news but declines if additionally exposed to an FBI report on threats from China. This suggests that the singling out of Chinese people, especially the Chinese government, in the rhetoric around COVID-19 and who to blame, may have hampered a sense of panethnic linked fate, and thus a collective political response, for other Asian Canadians.

Another interpretation is that East/Southeast Asians *had* felt a sense of linked fate with Chinese people, but this alone was not enough to politically mobilize the entire group. Recent work has emerged to interrogate the connection between linked fate and political action and suggests that several conditions matter. For instance, Smith et al., (2024) find that among Black Americans, racial group consciousness can lead to political action when the political activity in question is perceived as favourable for group outcomes, and whether individuals view the cost as worthy to bear. In the current context, East/Southeast Asians may not have viewed disaffiliation with the Conservatives as a meaningful way to response to the rise in anti-Asian racism, especially if they were not singled out by the Conservatives as much as Chinese were. Hence, for a panethnic group like Asian Canadians, a shared experience of marginalization does not guarantee collective political action; instead, the perception of marginalization and the cost–benefit ratio of the action may matter more.

One area in which Asian Canadians were more consistent was the shared growth in NDP support from the pre-2021 to 2021 federal election, but this was the case for the rest of the sample as well. South Asians experienced a slightly sharper increase in NDP support than other groups, which may explain in part their relatively larger decline in Conservative Party support. One potential reason for this is co-ethnic voting, since the NDP has been led since 2017 by Jagmeet Singh, a Sikh man and the first ever racialized leader of one of the three major Canadian political parties (Bouchard, 2022). As such, co-ethnic voting for South Asians may be a larger driver of the rejection of the Conservative Party than potential feelings of linked fate with Chinese people. In another way, Chinese voters may also be showing more support for the NDP due to co-ethnic voting which itself could be a signal of panethnic linked fate (Besco, 2015). However, given that the NDP is considered the weakest party of the three, there is a chance that voters may be voting NDP to spoil their vote (Caruana et al., 2015).

Regardless, that the decline in Conservative Party support is strongest among Chinese compared to non-Chinese Asian respondents is consistent with findings from another study that found that increased Democratic partisanship due to COVID-19-related anti-Asian racism was strongest for Chinese Americans, but less-so for Indian, Korean, or Vietnamese Americans (Chan et al., 2022a, 2022b). However, the current study’s findings do not negate the possibility of panethnic linked fate entirely; instead, for this vastly diverse group, linked fate and collective political action may be conditional upon more than exposure to group discrimination. The multiplicity and fluidity of ethnic identity for a panethnic group like Asian Canadians complicate canonical understandings of how social exclusion fosters political action. Hence, how panethnic identity influences people’s politics, especially among Asian Canadians, warrants further study. As Asians have often been conceptualized and analysed as a monolith, the study’s findings also underscore the need to more deliberately consider intragroup dynamics in examining racialized minority politics.

This study is not without limitations however, and several opportunities exist for future work in this area. First, due to having repeated cross-sectional data, I could not estimate within-person changes in political party preferences which means unobserved differences between the groups over time could account for some of the changes in party preferences. Another data limitation is that the CES does not explicitly ask respondents about experiences with discrimination, nor about panethnicity and panethnic linked fate. As such, I cannot measure discrimination exposure, nor conclude explicitly that discrimination dampened panethnicity or linked fate. Hence, my hypotheses and interpretation of the evidence are largely informed by existing literature on minority politics and voting choices as reactions to discriminatory political rhetoric. I also cannot disentangle analytically whether it is exclusion specifically at the level of political elites, and nowhere else, that induced changes in political party support. Political elites are theoretically a more upstream source of exclusion given the ability for elites to normalize racist attitudes (e.g., Chan et al., 2022a, 2022b) and so likely capture exclusion more fully than other sources. With the natural experiment approach however, I attempt to overcome these limitations albeit imperfectly with the observational data available. Future studies that utilize randomized experiments can isolate more precisely the mechanisms that link discrimination to panethnicity and voting preferences among Asian Canadians and other minoritized groups.

As well, the CES is only administered in English or French, which likely limited the Asian respondents in the analytical sample to those who were already strong in these languages. The implications are that this selection of Asian respondents may have had more understanding of and access to Canada’s political and media landscape, and thus, may have been more attuned to the political significance of this surge in racism compared to newcomers. While I control for immigration status, the language barrier may still have led to selection bias. The analytical sample also included the Permanent Residents who are not legally allowed to vote and the data are not nationally representative; hence, I cannot say whether these changes in party support precisely explain the actual 2021 federal election outcomes or whether they are generalizable to all Asian Canadians, though the results map onto other scholars’ interpretations of the election (e.g., Ellis, 2022; White, 2022). Lastly, while I attempted to capture the ethnic variation among the Asian Canadian diaspora, I had to create composite ethnic groups based generally on potential sensitivity to COVID-19-related anti-Asian racism. This was mainly for sample size, as together, my three Asian ethnic groups were only a total of 11.2% of the final CES sample. This method is far from perfect and future surveys that collect data on a larger sample of Asian Canadians are encouraged.

Still, this study contributes to the literature on racial group politics, especially with respect to the Asian diaspora, as I find that racist political discourse can alter political party loyalties, though primarily for those targeted in this racism. This paper responds to ongoing debates about what shapes linked fate among vastly diverse populations that are typically analysed as a monolith, like the Asian diaspora in western societies. Overall, societal awareness of racial injustice against Asian communities has arguably never been higher in recent history, due to COVID-19. This heightened, global concern underscores the urgent need to address the political needs of the Asian diaspora whose experiences of racism and injustice have been relatively obscured in public discourse and scholarship (Yip et al., 2021).

## Supplementary Information

Below is the link to the electronic supplementary material.

Supplementary file1 (PDF 960 KB)

## Data Availability

The publicly available data used in the study can be found at: Fournier, P., Cutler, F., Soroka, S., & Dietlind, S. (2015). *The 2015 Canadian Election Study* [Dataset]. https://ces-eec.arts.ubc.ca/english-section/surveys/ Stephenson, L. B., Harell, A., Rubenson, D., & Loewen, P. J. (2024a). *2019 Canadian Election Study (CES)—Online Survey* [Dataset]. Harvard Dataverse. 10.7910/DVN/DUS88V Stephenson, L. B., Harell, A., Rubenson, D., & Loewen, P. J. (2024b). *2019 Canadian Election Study (CES)—Phone Survey* [Dataset]. Harvard Dataverse. 10.7910/DVN/8RHLG1 Stephenson, L. B., Harell, A., Rubenson, D., & Loewen, P. J. (2024c). *2021 Canadian Election Study (CES)* (Version 3.1) [Dataset]. Harvard Dataverse. [10.7910/DVN/XBZHKC] (https:/doi.org/10.7910/DVN/XBZHKC).
