# Supplementary material for: How Xenophobia Shapes Political Party Support: Evidence from COVID-19 in Canada
Source: Race Soc Probl. 2025 Dec 2;18(1):9. doi: 10.1007/s12552-025-09480-y (PMC12672673; doi:10.1007/s12552-025-09480-y)
Supplement: Supplementary file 1 — Supplementary file1 (PDF 960 KB) [file 12552_2025_9480_MOESM1_ESM.pdf]

How xenophobia shapes political party support: Evidence from COVID-19 in Canada

Supplementary Tables

Table Supp 1. Difference-in-Differences estimates for changes in probability of reporting more support for redistribution from pre-2021 to 2021 2

Table Supp 2. Mediation Results for when redistribution preferences are treated as a mediator to explain the relationship between race/ethnicity and vote choice 2

Table Supp 3. Difference-in-differences estimates for vote choice when standard errors are clustered around treatment groups versus time period, Canada Election Study 2015-2021 2

Table Supp 4. Predicted probabilities voting for either of the three main political parties, Canada Election Study 2015-2021 (including missing demographic data) 2

Table Supp 5. Distribution of demographic characteristics and political variables by ethnicity group, Canadian Election Study 2015-2021 3

Supplementary Figures

Figure Supp 1. Predicted probability of reporting more support for redistribution by race/ethnicity 5

Figure Supp 2. Changes in the predicted probability of reporting more support for redistribution from pre-2021 to 2021 federal elections by race/ethnicity 5

Figure Supp 3. Change in the predicted probability of voting for the Conservative party from pre-2021 to 2021 federal elections by race/ethnicity 6

Figure Supp 4. Change in the predicted probability of voting for the Liberal party from pre-2021 to 2021 federal elections by race/ethnicity 6

Figure Supp 5. Change in the predicted probability of voting for the NDP from pre-2021 to 2021 federal elections by race/ethnicity 7

Figure Supp 6. Plot of interaction coefficients between ethnicity and year for vote choice, with 2015 as reference year and Other as control group, CES 2015-2021 7

Figure Supp 7. Plot of interaction coefficients between ethnicity and year for party ratings, with 2015 as reference year and Other as control group, CES 2015-2021 8

Figure Supp 8. Sensitivity analysis for violations of parallel trends for Conservative vote choice between Chinese and Others using the test of restriction of relative magnitude from *HonestDiD* 8

Figure Supp 9. Plot of interaction coefficients between ethnicity and year for vote choice, with 2019 as reference year and Other as control group, CES 2019-2021 9

Figure Supp 10. Plot of interaction coefficients between ethnicity and year for party ratings choice, with 2019 as reference year and Other as control group, CES 2019-2021 9

1. Supplementary Tables

Table Supp 1. Difference-in-Differences estimates for changes in probability of reporting more support for redistribution from pre-2021 to 2021

Comparison	Estimate	Std. Error	95% CI low	95% CI high
Chinese vs Other	0.0549*	0.000124	0.0547	0.0552
Non-Chinese ESEA vs Other	-0.0353*	0.001395	-0.0380	-0.0325
South Asian vs Other	-0.0123*	0.001086	-0.0144	-0.0101

Notes: Standard errors in parentheses; ESEA = East/Southeast Asian; CI = Confidence Interval; Party ratings are on a 100-point scale.
* *P < 0.001*

Table Supp 2. Mediation Results for when redistribution preferences are treated as a mediator to explain the relationship between race/ethnicity and vote choice

	Estimate	95% CI low	95% CI high	P-value
ACME	-0.00284	-0.00939	0.00	0.36
ADE	-0.02946	-0.04454	-0.02	<2e-16 ***
Total Effect	-0.03230	-0.04982	-0.02	<2e-16 ***
Proportion Mediated	0.08798	-0.10137	0.27	0.36

Notes: ACME = Average Causal Mediation Effects; ADE = Average Direct Effects

Table Supp 3. Difference-in-differences estimates for vote choice when standard errors are clustered around treatment groups versus time period, Canada Election Study 2015-2021

**Comparison**	**Conservative**		**Liberal**		**NDP**	
	Treatment	Time	Treatment	Time	Treatment	Time
Chinese vs. Other	-0.0827 (0.0019)***	-0.0827* (0.0021)	0.0887 (0.0007)***	0.0887* (0.0021)	-0.006 (0.0018)***	-0.006 (0.0043)
non-Chinese ESEA vs. Other	0.0041 (0.0025)	0.0041* (0.0009)	0.0168 (0.0017)***	0.0168 (0.0083)	-0.0209 (0.0013)***	-0.0209+ (0.0092)
South Asian vs. Other	-0.0349 (0.0015)***	-0.0349* (0.0003)	0.0245 (0.0006)***	0.0245* (0.0063)	0.0104 (0.0015)***	0.0104 (0.0065)

Table Supp 4. Predicted probabilities voting for either of the three main political parties, Canada Election Study 2015-2021 (including missing demographic data)

	Conservative	Liberal	NDP
Difference-in-Differences: Change in differences after pandemic			
Chinese vs. Other	-0.0822 (0.0024)***	0.0893 (0.002)***	-0.0071 (0.0043)
non-Chinese ESEA vs. Other	0.0017 (0.0009)	0.0175 (0.0084)*	-0.0192 (0.0093)*

South Asian vs. Other	-0.0362 (0.0004)***	0.0233 (0.0062)***	0.0129 (0.0066)
Pre-pandemic differences:			
Chinese vs. Other	0.1067 (0.0051)***	-0.0494 (0.0076)***	-0.0573 (0.0127)***
non-Chinese ESEA vs. Other	-0.0417 (0.0067)***	0.072 (0.0049)***	-0.0302 (0.0116)**
South Asian vs. Other	-0.0609 (0.0061)***	0.1135 (0.0059)***	-0.0526 (0.012)***
Constant	0.397 (0.0042)***	0.406 (0.0063)***	0.197 (0.0021)***

Notes: Standard errors in parentheses; ESEA = East/Southeast Asian.
*** *P < 0.001, ** P < 0.01, * P < 0.05*

Table Supp 5. Distribution of demographic characteristics and political variables by ethnicity group, Canadian Election Study 2015-2021

	**Chinese (N=1834)**	**Non-Chinese ESEA (N=1459)**	**SA (N=1810)**	**Other (N=40121)**	**Overall (N=45224)**
**Age**					
Mean (SD)	41.4 (15.0)	41.3 (16.1)	41.5 (16.4)	51.1 (16.9)	50.0 (17.1)
Median [min, max]	39.0 [18.0, 99.0]	39.0 [18.0, 98.0]	38.0 [18.0, 99.0]	53.0 [18.0, 99.0]	51.0 [18.0, 99.0]
**Gender**					
Man	889 (48.5%)	640 (43.9%)	862 (47.6%)	18530 (46.2%)	20921 (46.3%)
Woman	945 (51.5%)	819 (56.1%)	948 (52.4%)	21591 (53.8%)	24303 (53.7%)
**Employment**					
Working	1381 (75.3%)	1000 (68.5%)	1257 (69.4%)	22994 (57.3%)	26632 (58.9%)
Not working	190 (10.4%)	189 (13.0%)	223 (12.3%)	3562 (8.9%)	4164 (9.2%)
Retired	175 (9.5%)	160 (11.0%)	199 (11.0%)	11425 (28.5%)	11959 (26.4%)
Unemployed	75 (4.1%)	84 (5.8%)	104 (5.7%)	1492 (3.7%)	1755 (3.9%)
Other	13 (0.7%)	26 (1.8%)	27 (1.5%)	648 (1.6%)	714 (1.6%)
**Income**					
<30k	178 (9.7%)	201 (13.8%)	254 (14.0%)	5938 (14.8%)	6571 (14.5%)
30-60k	317 (17.3%)	270 (18.5%)	367 (20.3%)	8693 (21.7%)	9647 (21.3%)
60-90k	345 (18.8%)	337 (23.1%)	394 (21.8%)	8345 (20.8%)	9421 (20.8%)
90-110k	232 (12.7%)	151 (10.3%)	195 (10.8%)	3736 (9.3%)	4314 (9.5%)

	Chinese (N=1834)	Non-Chinese ESEA (N=1459)	SA (N=1810)	Other (N=40121)	Overall (N=45224)
>110k	535 (29.2%)	313 (21.5%)	367 (20.3%)	9406 (23.4%)	10621 (23.5%)
No answer	227 (12.4%)	187 (12.8%)	233 (12.9%)	4003 (10.0%)	4650 (10.3%)
**Education**					
Some HS or Less	43 (2.3%)	39 (2.7%)	57 (3.1%)	1727 (4.3%)	1866 (4.1%)
HS	131 (7.1%)	141 (9.7%)	163 (9.0%)	5884 (14.7%)	6319 (14.0%)
College	282 (15.4%)	293 (20.1%)	360 (19.9%)	12909 (32.2%)	13844 (30.6%)
Undergraduate	1015 (55.3%)	758 (52.0%)	798 (44.1%)	14342 (35.7%)	16913 (37.4%)
Postgraduate	363 (19.8%)	228 (15.6%)	432 (23.9%)	5259 (13.1%)	6282 (13.9%)
**Immigrant**					
Yes	1060 (57.8%)	821 (56.3%)	1083 (59.8%)	4644 (11.6%)	7608 (16.8%)
No	774 (42.2%)	638 (43.7%)	727 (40.2%)	35477 (88.4%)	37616 (83.2%)
**Vote choice**					
Liberal	735 (40.1%)	714 (48.9%)	978 (54.0%)	15907 (39.6%)	18334 (40.5%)
Conservative	744 (40.6%)	402 (27.6%)	442 (24.4%)	14731 (36.7%)	16319 (36.1%)
NDP	355 (19.4%)	343 (23.5%)	390 (21.5%)	9483 (23.6%)	10571 (23.4%)
**Conservative Party rating**					
Mean (SD)	51.2 (29.8)	46.1 (31.7)	43.2 (32.3)	45.3 (34.6)	45.5 (34.3)
Median [Min, Max]	60.0 [0, 100]	50.0 [0, 100]	42.0 [0, 100]	46.0 [0, 100]	49.0 [0, 100]
**Liberal Party rating**					
Mean (SD)	53.6 (28.2)	60.8 (28.9)	64.8 (29.0)	50.7 (33.3)	51.7 (32.9)
Median [Min, Max]	60.0 [0, 100]	69.0 [0, 100]	72.0 [0, 100]	60.0 [0, 100]	60.0 [0, 100]
**NDP rating**					
Mean (SD)	52.7 (26.3)	57.6 (26.2)	59.4 (27.3)	52.1 (29.9)	52.6 (29.6)
Median [Min, Max]	58.0 [0, 100]	61.0 [0, 100]	64.0 [0, 100]	59.0 [0, 100]	59.0 [0, 100]

2. Supplementary Figures

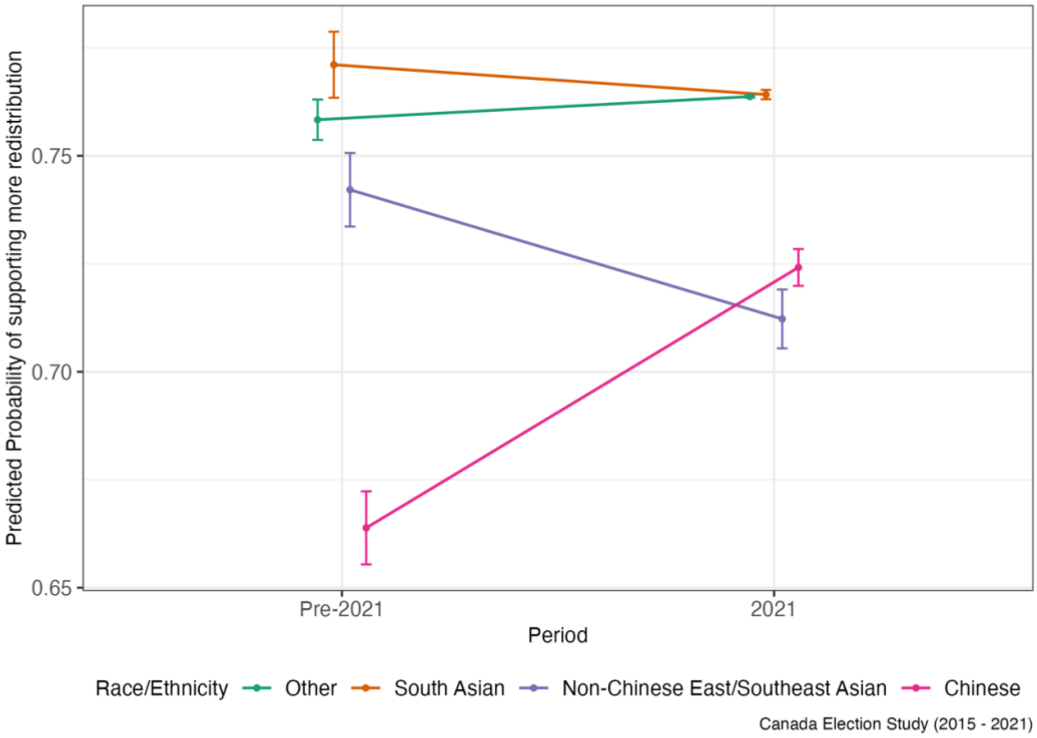

Figure Supp 1. Predicted probability of reporting more support for redistribution by race/ethnicity

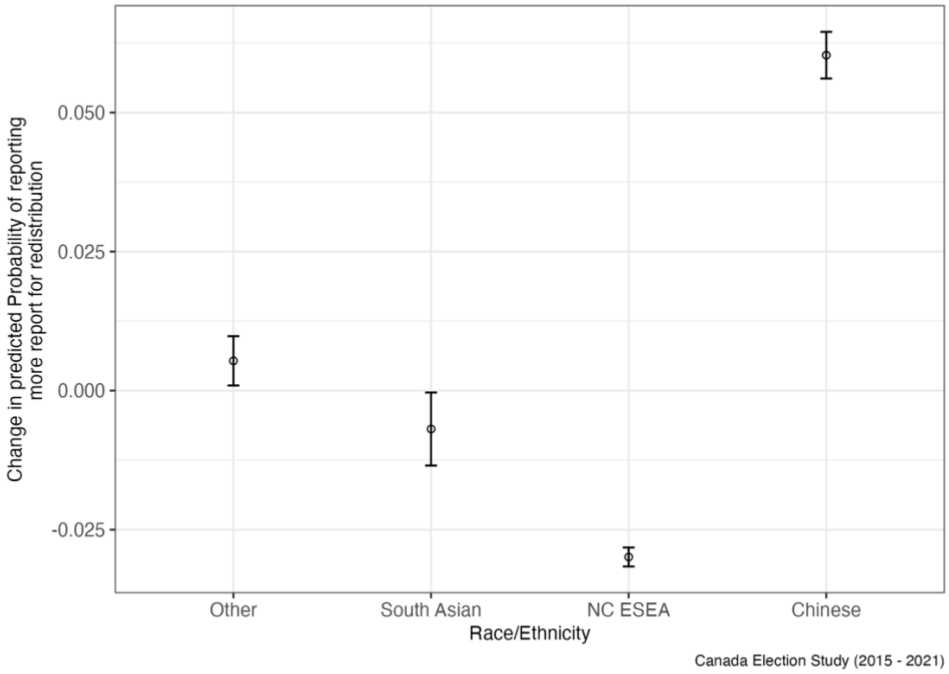

Figure Supp 2. Changes in the predicted probability of reporting more support for redistribution from pre-2021 to 2021 federal elections by race/ethnicity

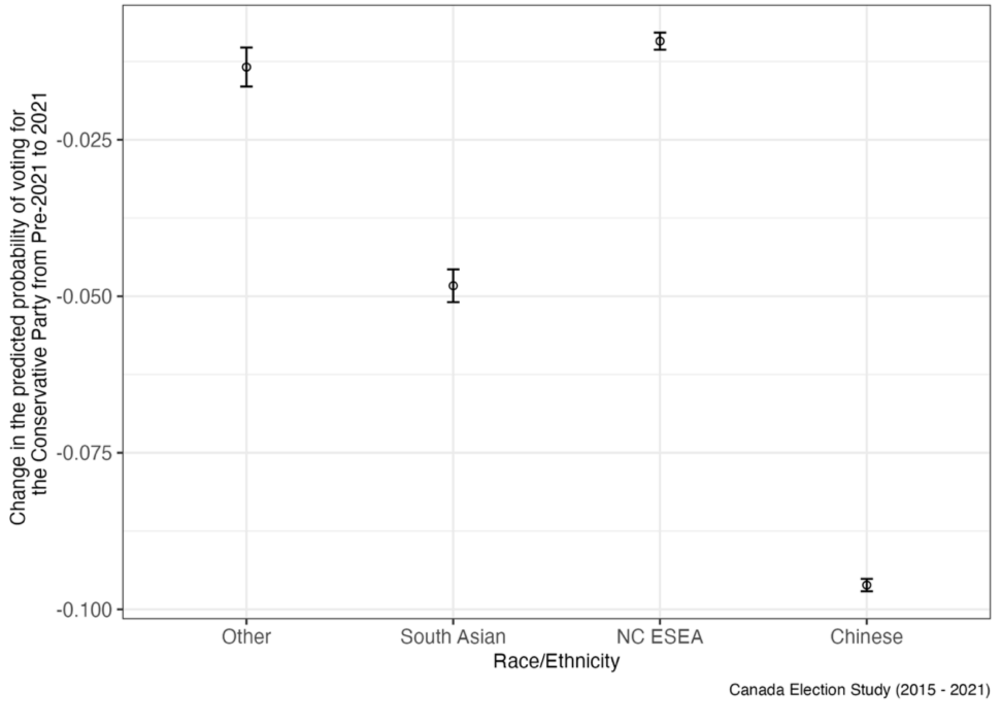

Figure Supp 3. Change in the predicted probability of voting for the Conservative party from pre-2021 to 2021 federal elections by race/ethnicity

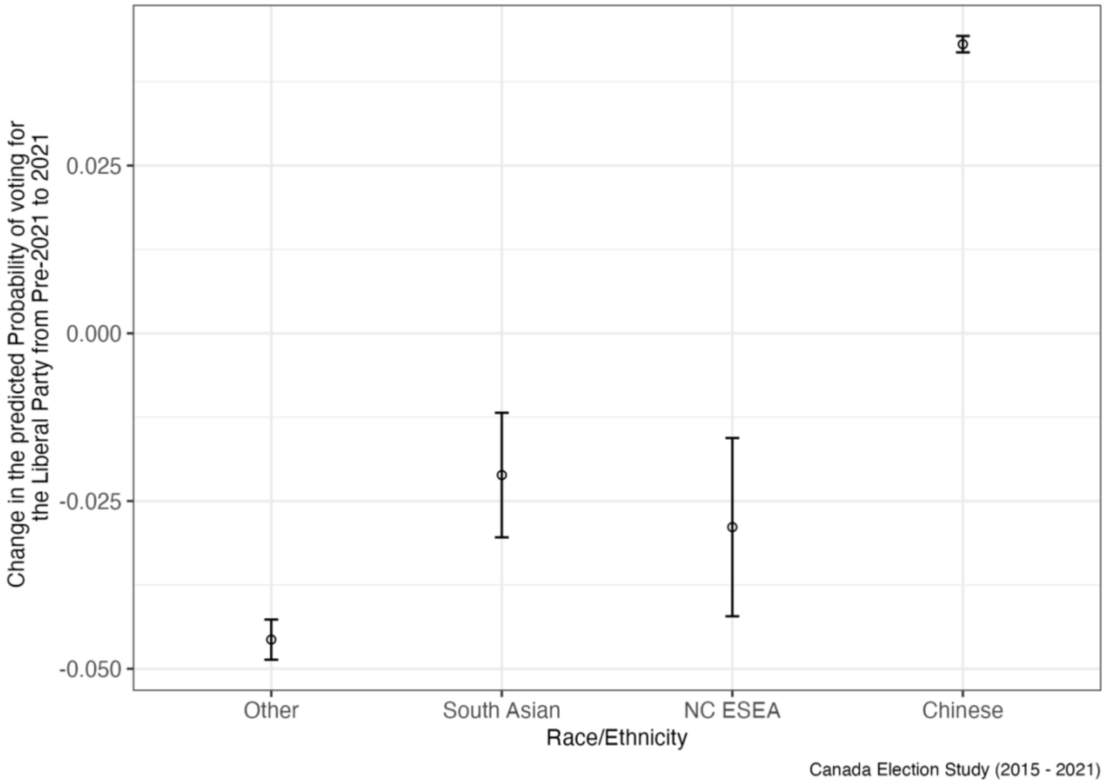

Figure Supp 4. Change in the predicted probability of voting for the Liberal party from pre-2021 to 2021 federal elections by race/ethnicity

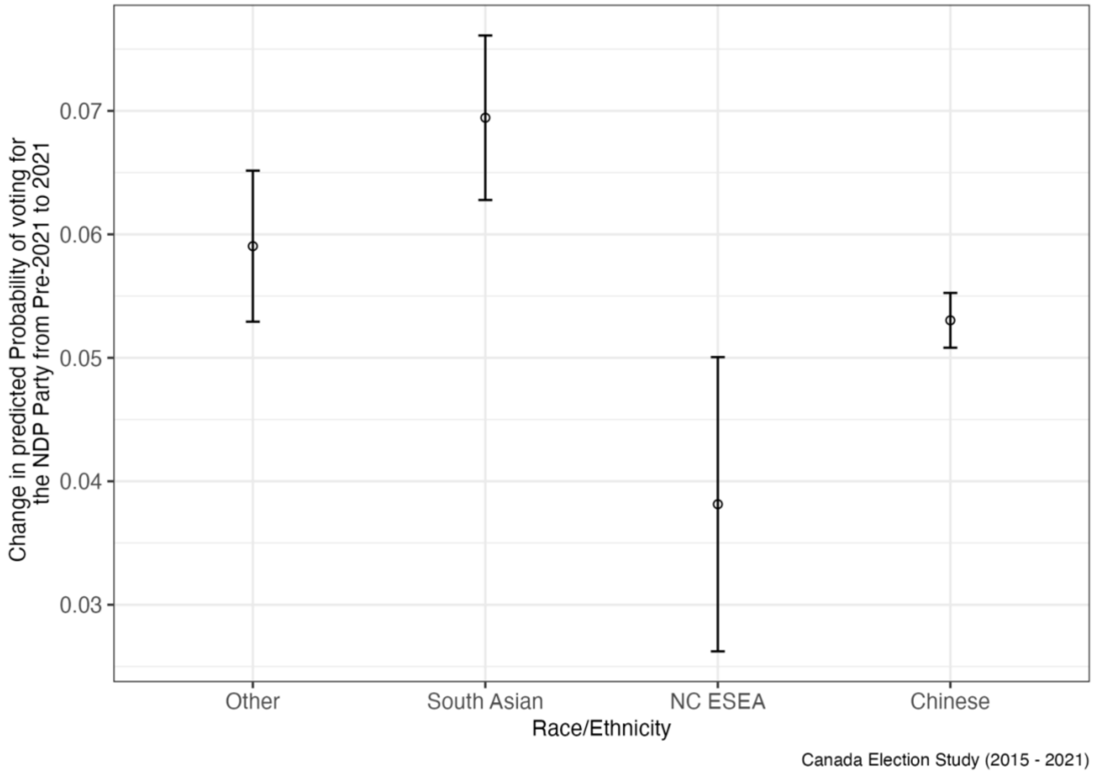

Figure Supp 5. Change in the predicted probability of voting for the NDP from pre-2021 to 2021 federal elections by race/ethnicity

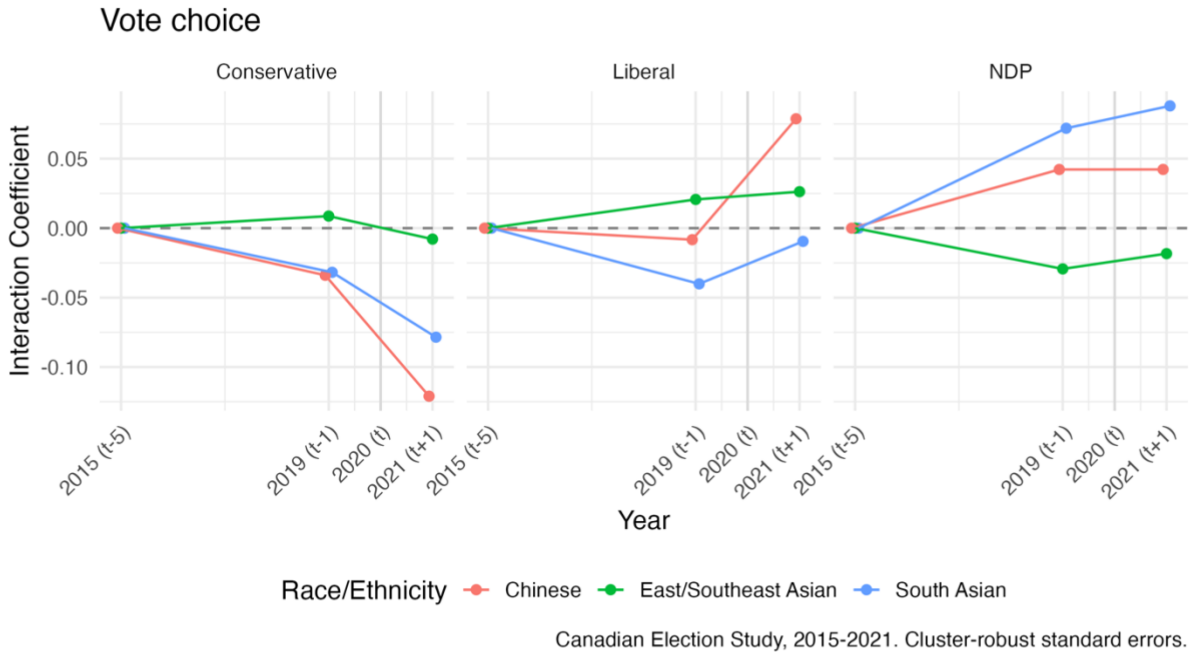

Figure Supp 6. Plot of interaction coefficients between ethnicity and year for vote choice, with 2015 as reference year and Other as control group, CES 2015-2021

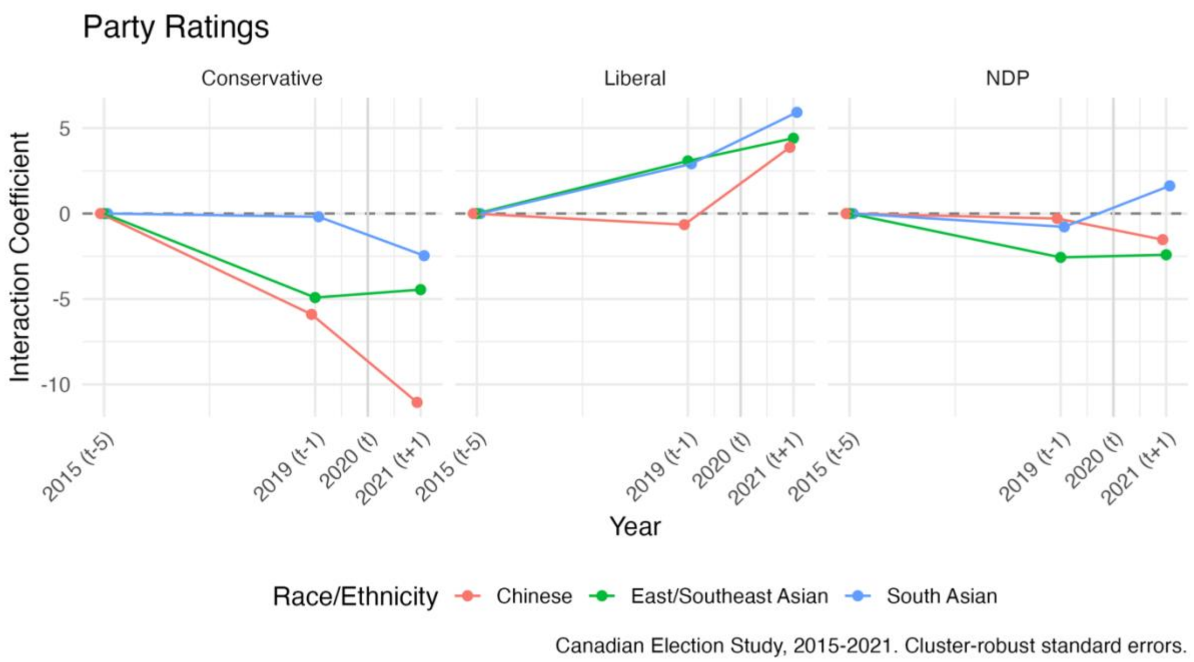

Figure Supp 7. Plot of interaction coefficients between ethnicity and year for party ratings, with 2015 as reference year and Other as control group, CES 2015-2021

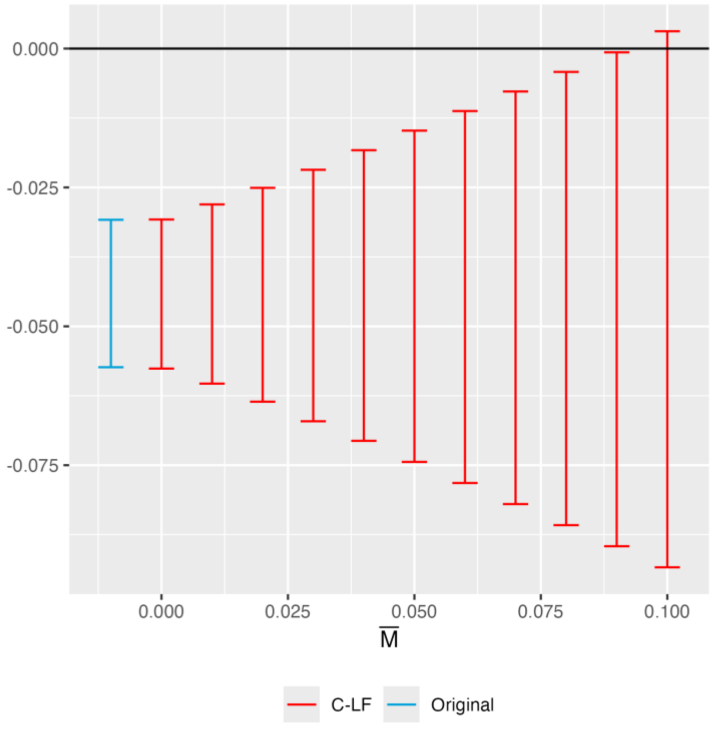

Figure Supp 8. Sensitivity analysis for violations of parallel trends for Conservative vote choice between Chinese and Others using the test of restriction of relative magnitude from *HonestDiD*

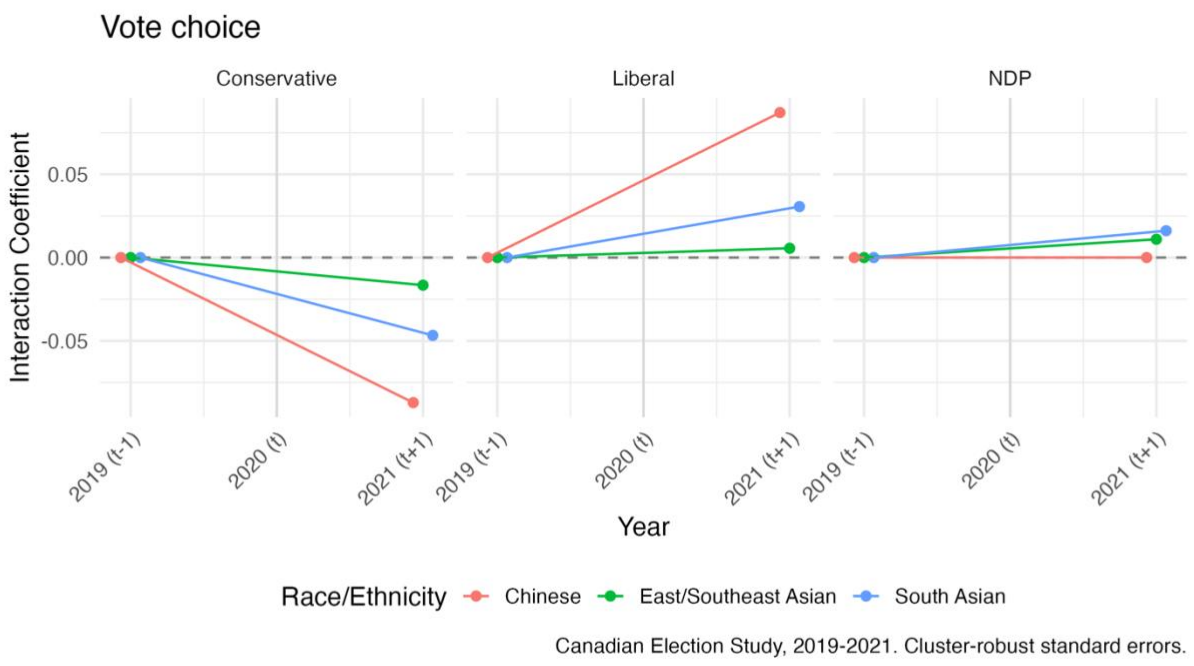

Figure Supp 9. Plot of interaction coefficients between ethnicity and year for vote choice, with 2019 as reference year and Other as control group, CES 2019-2021

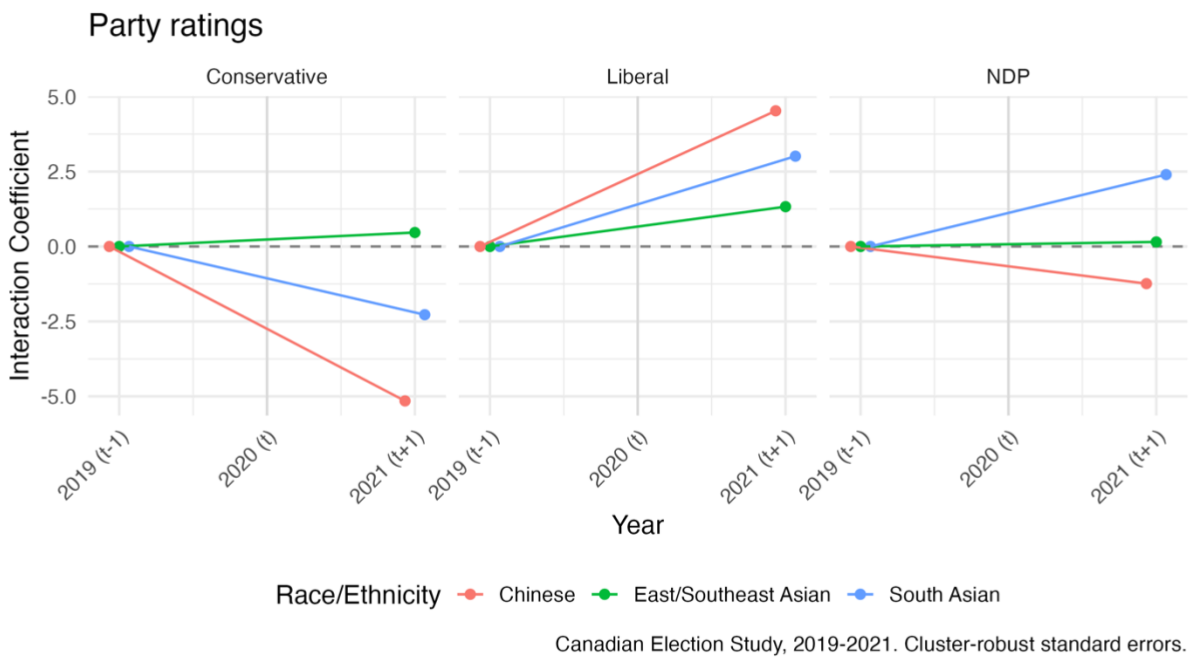

Figure Supp 10. Plot of interaction coefficients between ethnicity and year for party ratings choice, with 2019 as reference year and Other as control group, CES 2019-2021
